# Atypical audiovisual word processing in school-age children with a history of specific language impairment: an event-related potential study

**DOI:** 10.1186/s11689-016-9168-3

**Published:** 2016-09-05

**Authors:** Natalya Kaganovich, Jennifer Schumaker, Courtney Rowland

**Affiliations:** 1 Department of Speech, Language, and Hearing Sciences, Purdue University, Lyles Porter Hall, 715 Clinic Drive, West Lafayette, IN 47907-2038 USA; 2 Department of Psychological Sciences, Purdue University, 703 Third Street, West Lafayette, IN 47907-2038 USA

**Keywords:** Audiovisual matching, Specific language impairment, Lexical processing, Speech-in-noise perception, Event-related potentials

## Abstract

**Background:**

Visual speech cues influence different aspects of language acquisition. However, whether developmental language disorders may be associated with atypical processing of visual speech is unknown. In this study, we used behavioral and ERP measures to determine whether children with a history of SLI (H-SLI) differ from their age-matched typically developing (TD) peers in the ability to match auditory words with corresponding silent visual articulations.

**Methods:**

Nineteen 7–13-year-old H-SLI children and 19 age-matched TD children participated in the study. Children first heard a word and then saw a speaker silently articulating a word. In half of trials, the articulated word matched the auditory word (congruent trials), while in another half, it did not (incongruent trials). Children specified whether the auditory and the articulated words matched. We examined ERPs elicited by the onset of visual stimuli (visual P1, N1, and P2) as well as ERPs elicited by the articulatory movements themselves—namely, N400 to incongruent articulations and late positive complex (LPC) to congruent articulations. We also examined whether ERP measures of visual speech processing could predict (1) children’s linguistic skills and (2) the use of visual speech cues when listening to speech-in-noise (SIN).

**Results:**

H-SLI children were less accurate in matching auditory words with visual articulations. They had a significantly reduced P1 to the talker’s face and a smaller N400 to incongruent articulations. In contrast, congruent articulations elicited LPCs of similar amplitude in both groups of children. The P1 and N400 amplitude was significantly correlated with accuracy enhancement on the SIN task when seeing the talker’s face.

**Conclusions:**

H-SLI children have poorly defined correspondences between speech sounds and visually observed articulatory movements that produce them.

## Background

Speech perception is audiovisual in nature in the majority of daily situations. We notice this most easily when a noisy environment or hearing loss makes us focus on the speaker’s mouth (e.g., [5, 6, 44, 85, 100, 113]). However, listening to a non-native language or to speech with ambiguous content also benefits from seeing the talker’s face [67, 82].

Accumulating evidence suggests that sensitivity to visual speech cues emerges early in development (e.g., [50, 57]) and continues to mature throughout adolescence [4, 21, 49, 84, 105]. It facilitates the acquisition of important building blocks of language, such as phonemes [101] and words [43, 45], and shapes the development of both speech production [56] and speech perception [5, 54]. The fact that visual speech cues influence multiple aspects of typical language acquisition invites the question of whether impairment in the processing of visual articulatory movements and/or difficulty in integrating such movements with concurrent auditory speech may underlie some of the deficits observed in developmental language disorders, such as specific language impairment (SLI).

### Audiovisual speech perception in SLI

SLI is a language disorder that affects approximately 7 % of preschool children in the USA [104]. It is characterized by significant linguistic difficulties without an apparent cause, such as hearing impairment, frank neurological disorders, or low non-verbal intelligence [55]. Studies of audiovisual speech perception in SLI are few. The majority of them are based on the McGurk illusion [9, 39, 62, 71, 72]. In this well-known phenomenon, an auditory “pa” is typically dubbed onto an articulation of “ka.” The resultant perception of “ta” or “tha” is said to reflect audiovisual integration because the perceived phoneme represents a compromise between the bilabial auditory signal and the velar visual speech cues. Overall, studies of the McGurk illusion in SLI reported that children and adults with this language disorder have fewer illusory McGurk perceptions and fewer responses based on visual information only, suggesting that they are influenced significantly less than their TD peers by visual speech cues during audiovisual speech perception.

Although informative, McGurk studies have serious limitations. Because McGurk syllables provide conflicting auditory and visual cues to the phonemes’ identity, they may pose great difficulty to children with SLI, whose phonological processing is weaker than that of their TD peers (e.g., [40]). Additionally, a recent study by Erickson and colleagues reported that perception of the McGurk illusion and of the more natural audiovisually congruent speech engage distinct neural structures [26]. This finding is in agreement with other reports showing that different types of audiovisual tasks and stimuli activate at least somewhat disparate brain areas (e.g., [11, 97, 98]). Therefore, difficulty with the McGurk illusion in children with SLI cannot be generalized to the perception of more naturalistic congruent audiovisual speech.

One recent study did compare the ability of children with language learning impairment^1^ (LLI) and their age-matched and language-matched TD peers to perceive videos of a speaker articulating words and sentences [48]. Their tasks included lip-reading and speech-in-noise (SIN) perception (with the latter administered with and without the presence of the talker’s face). The authors found that the LLI children’s ability to identify individual *words* based on visual information improved with age in a manner similar to what was observed in their TD peers; however, their ability to identify *sentences* based on visual speech cues did not. Additionally, although LLI children of all ages benefited from the presence of the talker’s face when listening to SIN, they did so to a smaller degree than their TD peers.

In sum, previous studies show that at least some audiovisual skills are either impaired or weakened in SLI, but more studies with naturalistic stimuli are needed. Additionally, because the majority of studies on audiovisual processing in SLI have been behavioral, we know little about the sensory and/or cognitive mechanisms that underlie audiovisual speech perception difficulties in this population. Finally, and importantly, we also do not yet know whether audiovisual speech perception ability in SLI is related to overall language skills in this group, and if so, which aspects of linguistic competence show the closest connection with audiovisual processing.

### Why study children with a history of SLI?

Children are typically diagnosed with SLI when they are 4–5 years of age. However, in many cases, this is a life-long disorder, and the prognosis for children with SLI is often poor [70]. Importantly, multiple studies show that even those children who appear to be “recovered” in fact have milder but persistent deficits in a variety of language skills [18, 40, 70, 99]. Yet others seemingly recover early during development but begin to manifest deficits again during school years. Such re-appearance of deficits in older children led Scarborough and Dobrich to suggest that in many cases the recovery is only “illusory,” with high risk for these children to fall behind their peers again [88]. Standardized language tests are not always sensitive to subtler language difficulties of older children with SLI. Furthermore, scores within the normal range on such tests may not, by themselves, be sufficient to establish a true recovery because they may hide atypical cognitive strategies used by these children during testing [47]. Because eligibility for schools’ speech-language pathology services is typically determined by performance on standardized tests, many school-age children with SLI no longer qualify for language therapy. Yet, we know that compared to their TD peers, these children often have lower academic achievement [99], more social problems [31, 32, 75], and a higher risk of being diagnosed with attention deficit/hyperactivity (ADHD) disorder [65, 80, 81] and dyslexia [95].

School years place increased demands on children’s cognitive and linguistic abilities. In an academic setting, most learning happens in a face-to-face situation, in which audiovisual speech perception skills are of great value, especially if we take into account the high level of noise (~65 dB) in a typical school environment [93]. Because lip movements usually precede the onset of auditory speech (e.g., [17, 35, 108]), sensitivity to correspondences between lip movements and specific speech sounds may provide significant benefits by helping listeners formulate an expectation for the incoming auditory signal and facilitate phonological and lexical processing. Studies of younger children with SLI suggest that some aspects of audiovisual speech perception may be impaired in this disorder, but we do not know if by school-age audiovisual skills in this population are more similar to those of their TD peers. In this study, we examined audiovisual processing in children who were diagnosed with SLI when they were 4–5 years of age and who were 7–13 years of age at the time of the current testing. Their detailed characteristics are provided in the “Methods” section. Compared to their TD age-matched peers, they showed significantly weaker language skills as measured by the Clinical Evaluation of Language Fundamentals (CELF-4; [91]). However, most did not fall below the clinical cut-off of this test. We will therefore refer to this group as children with a history of SLI (H-SLI). Understanding how audiovisual speech perception functions in school-age H-SLI children may not only help identify academic strategies that are most effective for this group of children but also add an important dimension to our knowledge about SLI, which is typically studied within the context of the auditory modality only.

### Current study

#### General approach

We used a cross-modal repetition priming paradigm to test children's ability to match auditory words with observed visual articulations. School-age H-SLI children and their TD peers first listened to an auditory word referring to a common and familiar object (e.g., pumpkin) and then determined whether the following visual silent articulation matched the heard word (experiment 1). In half of all trials, the articulation matched the word (congruent trials), while in another half the articulation differed significantly from the auditory word during the initial syllable (incongruent trials). We combined this paradigm with event-related potential (ERP) recordings, which allowed us to evaluate different stages of visual processing, as described below. Additionally, in a separate experiment (experiment 2), we measured the degree to which seeing the talker’s articulating face facilitated perception of SIN in both groups of children. Last, through a series of multiple regressions, we examined which ERP measures of visual processing can predict (1) children’s overall linguistic ability and (2) children’s improvement on the SIN task when seeing the talker’s face.

#### Hypotheses

We have capitalized on the excellent temporal resolution of the ERP method in order to examine three distinct stages of visual processing of articulatory movements. First, difficulties in using visual speech cues may arise from atypical sensory encoding of visual information more generally. If such encoding is less robust in H-SLI children, the addition of visual speech cues to the auditory signal may not lead to significant improvement. To examine this possibility, we compared ERPs elicited by the static face of the speaker and by the pictures that accompanied auditory words (see “Methods” section) in the two groups of children. Both types of visual stimuli elicited a sequence of the visual P1, N1, and P2 components over occipital sites. These components are thought to be sensitive to different aspects of visual processing. We did not have an a priori prediction about specific visual components that may differ between H-SLI and TD children. Therefore, all three components were analyzed.

Second, reduced influence of articulatory movements on speech perception may also result from later phonological and lexical stages of processing. To examine this possibility, we compared ERPs elicited by congruent and incongruent articulations in order to isolate the N400 and the late positive complex (LPC) ERP components that index these two stages of linguistic analysis. The N400 component is most known for its sensitivity to semantic properties of words (such as the ease with which semantic representations may be accessed during perception (for reviews, see [24, 42, 51–53])). However, we capitalized on a different characteristic of this component—namely, in the context of priming tasks, the N400 amplitude is sensitive to phonological correspondences between prime and target words [78, 79], with greater negativity to phonological mismatches. Importantly, a study by Van Petten and colleagues demonstrated that the onset of the N400 component precedes the point at which words can be reliably recognized [106], suggesting that this component is elicited as soon as enough information has been processed to determine that the incoming signal mismatches the anticipated one. Therefore, we expected that the N400 modulation in our paradigm will reflect sub-lexical processing of the observed articulation, with greater N400 to incongruent articulations. We hypothesized that a reduction in the N400 amplitude in H-SLI children would suggest that they may have imprecise correspondences between speech sounds and the articulatory movements that produce them.

The LPC ERP component belongs to a family of relatively late positive deflections in the ERP waveform that varies in distribution and amplitude depending on the task used. Of particular relevance to our paradigm is the sensitivity of this component to word repetition (for reviews, see [30, 87]). More specifically, the LPC is larger to repeated as compared to not repeated words (e.g., [69, 74]), suggesting that it indexes some aspects of the recognition process. Accordingly, we expected a larger LPC component to congruent than incongruent articulations, reflecting recognition of a word silently mouthed by the talker. We hypothesized that a reduction in the LPC amplitude in H-SLI children would suggest that they have weaker associations between auditory words and the *sequences* of articulatory gestures that produce them.

Taken together, our analyses allowed us to compare brain responses in TD and H-SLI children during complex visual encoding, phonological audiovisual matching, and word recognition stages of visual speech perception and to examine which of these ERP indices relate to children’s linguistic ability and the degree of benefit gained from audiovisual speech.

## Methods

### Participants

Nineteen children with a history of SLI (H-SLI) (5 female; mean age 10;0; range 7;7–13;8) and 19 children with typical development (TD) age-matched within 5 months to the H-SLI children (7 female; mean age 10;0; range 7;3–13;7) participated in the study. All gave their written consent to participate in the experiment. The study was approved by the Institutional Review Board of Purdue University (protocol # 0909008484), and all study procedures conformed to The Code of Ethics of the World Medical Association (Declaration of Helsinki) (1964).

H-SLI children were originally diagnosed with SLI during preschool years (3;11–5;9 years of age) based on either the Structured Photographic Expressive Language Test—2nd Edition (SPELT-II, [112]) or the Structured Photographic Expressive Language Test—Preschool 2 (SPELT-P2; [20]). One additional H-SLI child was diagnosed based on the Clinical Evaluation of Language Fundamentals Preschool—2nd edition (CELF-P2; [92]). All tests have shown good sensitivity and specificity [36, 77]. Children diagnosed with SPELT-P2 (*n* = 13) received the standard score of 86 or less (mean 76, range 61–86, SD = 8.4). According to the study by Greenslade and colleagues [36], the cut-off point of 87 provides good sensitivity and specificity for the tested age range. All children’s standard scores on SPELT-P2 fell below the 24th percentile (mean 10, range 2–23, SD = 7). Children diagnosed with SPELT-II (*n* = 5) received raw scores of 18–26, all of which fell below the 5th percentile. Finally, the child diagnosed with CELF-P2 received a standard score of 79. In sum, the H-SLI children showed significant language impairment at the time of the diagnosis. All but one of the H-SLI children had received some form of language therapy in the years between the original diagnosis of SLI and the current study (mean of 5 years, range 2.5–8 years, SD = 1.77), with eight H-SLI children still receiving therapy at the time of this study.

We administered four subtests of CELF to all children in order to assess their current language ability—the Concepts and Following Directions (C&FD, 7–12 year olds only), Recalling Sentences (RS), Formulated Sentences (FS), Word Structure (WS, 7 and 8 year olds only), Word Classes-2 Total (WC-2, 9–12 year olds only), and Word Definitions (WD, 13 year olds only). Taken together, these subtests yielded the Core Language Score (CLS), which reflects general linguistic aptitude. Additionally, we evaluated children’s verbal working memory with the non-word repetition test [23] and the Number Memory Forward and Number Memory Reversed subtests of the Test of Auditory Processing Skills—3rd edition (TAPS-3; [60]). All children were administered the Test of Nonverbal Intelligence—4th edition (TONI-4; [10]) to rule out intellectual disability and the Childhood Autism Rating Scale—2nd edition [89] to rule out the presence of autism spectrum disorders. The level of mothers’ and fathers’ education was measured as an indicator of children’s socio-economic status (SES). The level of risk for developing ADHD was evaluated with the help of the short version of the Parent Rating Scale of the Conners’ Rating Scales—Revised [16]. In all participants, handedness was assessed with an augmented version of the Edinburgh Handedness Questionnaire (M. S. [15, 73]).

Seven H-SLI children had a current diagnosis of attention deficit/hyperactivity disorder (ADHD), with four taking medications to control symptoms. Because ADHD is highly comorbid with SLI [65] and because language difficulties associated with ADHD proper are at least partially different from the language difficulties associated with SLI [80, 81], we did not exclude these children from our sample. Additionally, one H-SLI child had a diagnosis of dyslexia.^2^ None of the TD children had any history of atypical language development, ADHD, or reading difficulties. All participants were free of neurological disorders (e.g., seizures), passed a hearing screening at a level of 20 dB HL at 500, 1000, 2000, 3000, and 4000 Hz and reported to have normal or corrected-to-normal vision. Three children in the H-SLI group and two children in the TD group were left-handed. All other participants were right-handed.

### Experiment 1—audiovisual matching task

#### Stimuli

Stimuli for experiment 1 consisted of auditory words, silent videos of their articulations, and pictures matching words’ meanings. We used 96 words from the MacArthur Bates Communicative Developmental Inventories (Words and Sentences) [27] as stimuli. All words contained 1-2 morphemes and were 1 to 2 syllables in length with two exceptions – “elephant” and “teddy bear.” Words contained between 1 and 8 phonemes, with diphthongs counted as 1 phoneme. Words were produced by a female speaker and recorded with a Marantz digital recorder (model PMD661) and an external microphone (Shure Beta 87) at a sampling rate of 44,100 Hz. Sound files were edited in the Praat software [8] so that the onset and offset of sound were preceded by 50 ms of silence. Final sound files were root-mean-square normalized to 70 dB.

Videos showed a female talker dressed in a piglet costume articulating one word at a time. The costume made it easier to turn the paradigm into a game and to maintain children’s attention. The actor’s mouth area was left free of makeup except for bright lipstick and did not obscure natural muscle movements of the lower face during articulation. The videos’ frame per second rate was 29.97. The audio track of the video recording was removed in Adobe Premier Pro CS5 (Adobe Systems Incorporated, USA). Articulation portions of videos ranged from 1133 ms (for “car”) to 1700 ms (for “sandbox”).

Each of the words was matched with a color picture from the Peabody Picture Vocabulary Test (pictures were used with the publisher’s permission) [25] that exemplified the word’s meaning (for example, a picture of toys was matched with the word “toys”). Pictures served as fixation points to better maintain children’s attention on the computer monitor and minimize eye movements.

#### Experimental design

**Fig. 1:**
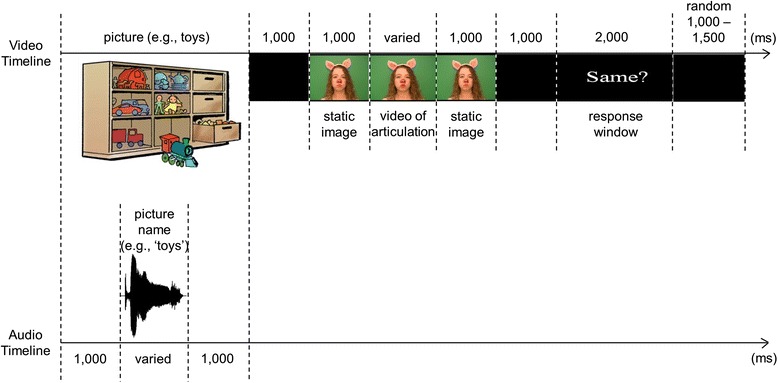
Schematic representation of a trial in the audiovisual matching task. Note that separate timelines are shown for the video and audio tracks. The video of articulation was congruent in half of all trials (e.g., participants saw the piglet silently articulate “toys” after hearing “toys” at the start of the trial) and incongruent in the other half of trials (e.g., participants saw the piglet silently articulate “bus” after hearing “toys” at the start of the trial). The onset of articulation was used as time 0 for the N400, LPC, and anterior negativity ERP averages. The onset of the pictures was used at time 0 for the visual P1, N1, and P2 ERP averages elicited by pictures, while the onset of the static picture of the talker’s face, prior to the onset of articulation, was used as time 0 for the visual P1, N1, and P2 ERP averages elicited by the talker’s face. This figure was originally published in [46]

The experimental design was identical to that described in an earlier study from our laboratory [46]. Each trial consisted of the following events (see Fig. 1). Participants saw a color picture of a common object/person (e.g., toys, mailman). While the image was on the screen, participants heard the object named (e.g., they heard a female speaker pronounce the word “toys” or “mailman”). A blank screen followed for 1000 ms. Next, a video of a female talker was presented. It consisted of a static image of the talker’s face taken from the first frame of the video (1000 ms), followed by a silent articulation of a word, followed by the static image of the talker’s face taken from the last frame of the video (1000 ms). In half of all trials, the talker’s articulation matched the previously heard word (*congruent* trials; for example, participants saw the talker articulate “toys” after hearing the word “toys”), while in another half, the talker’s articulation clearly mismatched the previously heard word (*incongruent* trials; for example, participants saw the talker say “bus” after hearing the word “toys”). The appearance of the screen with “Same?” written across it signaled the start of the response window. It lasted 2000 ms, during which participants had to determine whether the silently articulated word was the same as the word they heard at the beginning of the trial. Trials were separated by a temporal period randomly varying between 1000 and 1500 ms. Responses were collected via a response pad (RB-530, Cedrus Corporation), with the response hand counterbalanced across participants. Stimulus presentation and response recording was controlled by the presentation program (https://www.neurobs.com/).

Each participant completed 96 trials (48 congruent and 48 incongruent). For incongruent trials, 48 pairs of auditory and silently articulated words were created such that their visual articulation differed significantly during the word onset. In most cases (35 out of 48 pairs), this was achieved by pairing words, in which the first consonants differed visibly in the place of articulation (e.g., belt vs. truck). In 6 pairs, the first vowels of the words differed in the shape and the degree of mouth opening (e.g., donkey vs. candy). In the remaining 7 pairs, the first sounds were a labial consonant in one word (i.e., required a mouth closure (e.g., pumpkin)) and a vowel (i.e., required a mouth opening (e.g., airplane)) in another word. Heard and articulated words in incongruent pairs had no obvious semantic relationship. Two lists containing 48 congruent and 48 incongruent heard vs. articulated word presentations were created such that articulations that were congruent in list A were incongruent in list B. As a result, across all participants, we collected responses to the same articulations, which were *perceived* as either congruent or incongruent. Such counterbalancing also allowed for the control of word frequency, length, and complexity across congruent and incongruent trials. Lastly, 10 different versions of list A and 10 different versions of list B were created by randomizing the order of 96 trials. Each participant completed only one version of one list (e.g., participant 1 did list A version 1; participant 2 did list B version 1; participant 3 did list A version 2, participant 4 did list B version 2) Version 1 of lists A and B is shown in the Appendix. This task was combined with ERP recordings (see below).

In order to determine how many of the silent articulations could be recognized by our participants on incongruent trials and to evaluate their lip-reading abilities (which are often thought to contribute to SIN perception), we selected 20 silent articulations from the list of 96 used and asked each participant (in a separate session) to provide their best guess as to what word they thought the speaker was producing. The list of 20 words used for this task is shown in Table 1. In order to select words that reflected the diversity of lexical items used for the main task, this set of words included both one- and two-syllable words and contained items that started with either a labial (closed mouth) or an alveolar (open mouth) sound. No cues to the words’ identity were provided. This task is referred to henceforth as the lip-reading task. Because in many cases multiple auditory words may map onto similar observable articulatory movements, children were given credit not only for identifying the word that was in fact produced by the talker but also for reporting words that shared the same articulation with the target word. For example, words “Bob,” “Mom,” and “pop” were accepted as correct when children viewed the articulation of “mop.”

**Table 1:** Words presented during the lip-reading task

Bilabial/labiodental onset		Alveolar onset	
One-syllable words	Two-syllable words	One-syllable words	Two-syllable words
Boy	Pumpkin	Dog	Necklace
Mop	Mailman	Tree	Donkey
Farm	Window	Lamb	Sweater
Beach	Balloon	Knife	Zipper
Woods	Flower	Scarf	Teacher

#### ERP recordings and data analysis

##### General procedure

During the audiovisual matching task, the electroencephalographic (EEG) data were recorded from the scalp at a sampling rate of 512 Hz using 32 active Ag-AgCl electrodes secured in an elastic cap (Electro-Cap International Inc., USA). Electrodes were positioned over homologous locations across the two hemispheres according to the criteria of the International 10-10 system [2]. The specific locations were as follows: midline sites Fz, Cz, Pz, and Oz; mid-lateral sites FP1/FP2, AF3/AF4, F3/F4, FC1/FC2, C3/C4, CP1/CP2, P3/P4, PO3/PO4, and O1/O2; and lateral sites F7/F8, FC5/FC6, T7/T8, CP5/CP6, and P7/P8; and left and right mastoids. EEG recordings were made with the Active-Two System (BioSemi Instrumentation, Netherlands), in which the Common Mode Sense (CMS) active electrode and the Driven Right Leg (DRL) passive electrode replace the traditional “ground” electrode [64]. Data were referenced offline to the average of the left and right mastoids. The Active-Two System allows EEG recording with high impedances by amplifying the signal directly at the electrode [7, 63]. In order to monitor for eye movement, additional electrodes were placed over the right and left outer canthi (horizontal eye movement) and below the left eye (vertical eye movement). Prior to data analysis, EEG recordings were filtered between 0.1 and 30 Hz. Individual EEG records were visually inspected to exclude trials containing excessive muscular and other non-ocular artifacts. Ocular artifacts were corrected by applying a spatial filter (EMSE Data Editor, Source Signal Imaging Inc., USA) [76]. ERPs were epoched starting at 200-ms pre-stimulus and ending at 1800-ms post-stimulus onset. The 200 ms prior to the stimulus onset served as a baseline.

##### ERP components measured

We compared the peak amplitude and peak latency of the visual P1 (106–184 ms), N1 (164–248 ms), and P2 (264–370 ms) components elicited by pictures that accompanied auditory words and by the image of the talker's face. During the first 1000 ms of the video, the talker’s face was simply a static picture. Therefore, these early visual components do not reflect the encoding of articulatory movements. Presentation of pictures started 1000 ms prior to the onset of auditory words. This allowed us to measure visual ERPs to pictures without contamination by auditory processing. Measurement windows for visual P1, N1, and P2 were centered on each component’s peak over occipital sites (O1, OZ, O2), based on group averages. Each window was checked against individual files. For the analysis of visual components elicited by the talker’s face, the mean of 86 trials was available for the H-SLI group (SD = 6.86, range 71–96) and the mean of 89 trials for the TD group (SD = 4.21, range 81–96). For the analysis of visual components elicited by pictures, the corresponding numbers were 81 trials for the H-SLI group (SD = 7.8, range 58–91) and 82 trials for the TD group (SD = 8.1, range 68–93).

The onset of articulation elicited clear N400 and LPC. Additionally, although not predicted, both group and condition comparisons revealed a significant anterior negativity over the frontal scalp. These components’ mean amplitudes were measured over the following windows: 380–630 ms for N400, 930–1540 ms for LPC, and 1040–1470 ms for anterior negativity. N400 and LPC were measured over the CP, P, PO, and O sites. Anterior negativity was measured over the FP, AF, F and FC sites.

The window for the N400 component was based on earlier N400 studies in school-age children (e.g., [59, 110]) and the visual inspection of the grand average waveforms. Latencies of LPC and anterior negativity vary significantly from study to study. In order to select their measurement windows more objectively, we adopted the following procedure, based on suggestions by Groppe and colleagues ([37]; S. J.). We down-sampled individual averages to 100 Hz, which yielded 1 measurement per 10 ms of recording. We then selected one site over which the component of interest was most prominent (Pz for the LPC, AF3 for anterior negativity) and conducted a series of *t* tests on consecutive data points from the visual onset of each component until the end of the epoch (1800-ms post-stimulus onset). For the LPC component, *t* tests compared ERP responses to congruent and incongruent articulations in the TD group, while for the anterior negativity *t* tests compared TD and H-SLI groups’ ERPs to congruent articulations (the comparison in which the anterior negativity was most obvious). To control for type I error due to multiple comparisons, we used the false discovery rate (FDR) correction with the family-wise error set to 0.05. All consecutive points that survived the FDR correction^3^ formed the windows during which mean amplitudes of the LPC and anterior negativity were consequently measured over a larger array of electrodes (930–1540 ms for LPC and 1040–1470 ms for anterior negativity). In the H-SLI group, an average of 42 clean trials (SD = 3.7, range 33–46) were collected from each participant in the congruent condition and 43 (SD = 4, range 31–48) in the incongruent condition for the analysis of N400, LPC, and anterior negativity. In the TD group, the corresponding numbers were 45 trials (SD = 2.1, range 41–48) in the congruent and 43 (SD = 3.7, range 33–48) in the incongruent condition. For each participant, the number of available ERP trials for congruent and incongruent conditions was very comparable and differed on average by only 2.5 trials (SD = 2.15, range 0–9).

### Experiment 2—speech-in-noise (SIN) perception

#### Stimuli

In the second experiment, participants listened to the same 96 words used in the audiovisual matching task. However, this time words were embedded in a two-talker babble masker. The masker consisted of two female voices reading popular children’s stories. One sample was 3 min and 8 s long (by talker 1), and the other was 3 min and 28 s long (by talker 2). Both samples were manually edited in Praat to remove silent pauses greater than 300 ms and then repeated without discontinuity. The streams from the two talkers were root-mean-square normalized to 75 dB, mixed, and digitized using a resolution of 32 bits and a sampling rate of 24.414 kHz. Because 96 target words were root-mean-square normalized to 70 dB, the final stimuli had a −5-dB signal-to-noise ratio.

#### Experimental design

**Fig. 2:**
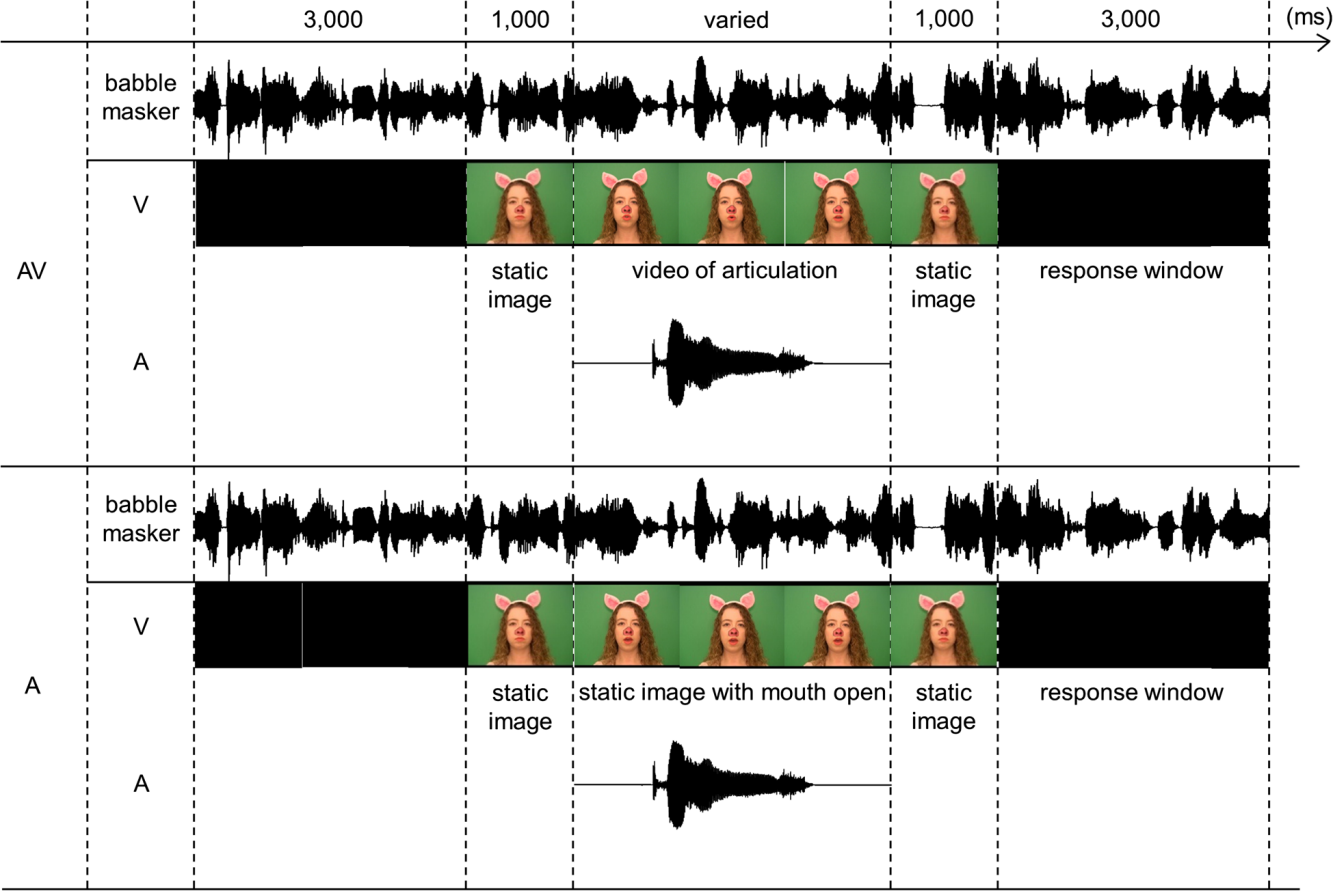
Schematic representation of a trial in the speech-in-noise (SIN) task. The SIN task had two conditions—the audiovisual (AV, *top panel*) and the auditory only (A, *bottom panel*). In the AV condition, participants saw a video of the piglet articulating target words. In the A condition, the video portion was replaced with a static image of the piglet’s face with her mouth open. The appearance of the open mouth picture in the A condition cued the participants to the fact that the onset of the auditory word is imminent but provided no visual speech cues to its identity. This figure was originally published in [46]

A schematic representation of the SIN trial is shown in Fig. 2. This task had two conditions—auditory only (A) and audiovisual (AV)—which were administered on two separate days. The order of A and AV conditions was counterbalanced across participants, but each participant completed both. The babble masker started 3 s prior to the first trial and was presented continuously until the end of the experiment. In the AV condition, participants saw videos of a talker producing each of 96 words. Each video was preceded and followed by a static image of a talker with a closed mouth, which lasted for 1000 ms. In the A condition, the same static images of the talker were present; however, the video portion was replaced with an image of the talker with her mouth open (see Fig. 2). The appearance of the open mouth picture in the A condition cued participants to the onset of the target auditory word, without providing any visual cues to its identity. Previous research shows that visual cues that reliably predict the onset of the auditory signal significantly improve the latter’s detection threshold [102]. The inclusion of the cue to the target word onset in the A condition aimed to make the attentional demands of the A and AV conditions more similar. Word presentations in both conditions were separated by 3 s, during which participants provided their verbal response about what they had heard. When unsure, participants were encouraged to give their best guess or to say “I don’t know.”

### Sequence of testing sessions

All testing occurred over three sessions administered on three different days. One of the SIN conditions (either A or AV) was administered during the first session, the audiovisual matching task and its lip-reading component—during the second session (with the lip-reading task always preceding the audiovisual matching task), and the second SIN condition—during the third session. Because the same words were used in the audiovisual matching task and in the SIN task, most participants’ sessions were separated by at least 7 days to minimize the possible effect of stimulus repetition.

### Statistical analyses

#### Behavioral and ERP measures

One-way ANOVA tests were used to compare group means on all screening tests. The homogeneity of variances across groups was evaluated with the Levene statistic. When variances differed, the Brown-Forsythe correction was applied. In all such cases, the corrected degrees of freedom and *p* value are reported. According to Cohen [14], in the case of one-way ANOVAs with two groups, each group needs 26 participants to detect a large effect with the power of 0.8 and the alpha level of 0.05. Since we had only 19 participants in each group, our negative results might have been due, in part, to insufficient power.

Repeated measures ANOVAs were used to determine whether groups differed in the number of correct responses, incorrect responses, misses, and in reaction time during the audiovisual matching task and to evaluate whether the SIN accuracy was higher in the AV compared to the A condition. Because the SIN task was completed by each child twice, we entered the sessions’ order as a between-subject variable to rule out its influence on the outcome. Repeated measures ANOVAs were also used to evaluate ERP components. When omnibus ANOVA analysis produced a significant interaction, it was further analyzed with step-down ANOVAs, with factors specific to any given interaction. When the assumption of sphericity was violated, we used the Greenhouse-Geisser adjusted *p* values to determine significance. Effect sizes, indexed by the partial eta squared statistic (*η*_*p*_^*2*^), are reported for all significant repeated measures ANOVA results. According to Cohen [13], we needed 26 participants in each group in order to detect a large effect in these factors with the alpha level of 0.05 and the power of 0.8. Twenty participants in each group would yield the power of 0.7.

#### Regressions

One of the main goals of this study was to understand a relationship between ERP measures of visual articulatory processing and (1) children’s linguistic ability and (2) children’s gains during audiovisual SIN perception. To this end, we conducted a series of stepwise multiple regression analyses, in which ERP measures were always entered as predictors and behavioral measures as outcomes. The ERP measures used for regressions were the average of the P1 peak amplitude to the talker’s face over O1, OZ, and O2 sites (this was the only visual component that differentiated the two groups of children, see “Results” section) and the N400 and LPC difference measures between congruent and incongruent trials averaged across all sites showing the effect of congruency. Behavioral measures included standard scores on the RS, FS, and C&FD subtests of CELF-4 (which were administered to children of all ages), accuracy on 4-syllable non-words (which showed the largest group difference), the degree of improvement on the SIN task when seeing the talker’s face (i.e., accuracy in the AV condition minus accuracy in the A condition), and accuracy on congruent and incongruent trials of the audiovisual matching task. To increase power, all regressions were conducted on the entire group of children (*n* = 38). To screen for outliers, we used the standardized DFBeta function in the SPSS Statistics program. Cases with the standardized DFBeta values over 1 have a significant influence over the regression model and are considered outliers [28]. Based on this threshold, one H-SLI child was excluded from the regression analysis between ERP measures and accuracy on incongruent trials of the audiovisual matching task. According to Cohen [14], a multiple regression analysis with three independent variables requires 34 participants to detect a large effect with the power of 0.8 and the alpha level of 0.05. Since we had over 34 participants in each regression analysis, we had enough power to detect only strong effects.

## Results

### Groups’ characteristics

Tables 2 and 3 contain group means and standard errors for all of the language, non-verbal intelligence, memory, and attention measures of the H-SLI and TD children. The two groups did not differ in either age, *F*(1,37) < 1, non-verbal intelligence, *F*(1,37) < 1, or SES as measured by mothers’ years of education, *F*(1,37) < 1. The fathers of H-SLI children had on average 3.4 years of education less than the fathers of TD children. This difference was statistically significant, *F*(1,31) = 12.73, *p* = 0.001. Information on fathers’ years of education was not available for three children in the TD group and three children in the SLI group. The ADHD Index and CARS scores also showed small but significant group differences, with higher (i.e., less typical) scores in the H-SLI group: ADHD Index, *F*(1,27) = 6.629, *p* = 0.016; CARS, *F*(1,23) = 5.199, *p* = 0.032.

**Table 2:** Group means for age, non-verbal intelligence (TONI-4), presence of autism (CARS-2), socio-economic status (parents’ education level), and linguistic ability (CELF-4)

	H-SLI	TD	*F*	*p*
Age (years; months)	10;0 (0.4)	10;0 (0.4)	<1	0.988
TONI-4	106.8 (2.1)	109.3 (2.3)	<1	0.43
CARS-2	15.8 (0.3)	15.1 (0.1)	5.199	0.032
Mother’s education (years)	15.4 (0.8)	15.5 (0.5)	<1	0.916
Father’s education (years)	13.8 (0.6)	17.1 (0.7)	12.73	0.001
CELF-4				
CF&D	9.6 (0.5)	12.1 (0.4)	13.338	0.001
RS	7.8 (0.5)	12.1 (0.5)	38.619	<0.001
FS	9.7 (0.4)	12.8 (0.3)	36.818	<0.001
WS	9.5 (1.1)	11.4 (0.5)	2.177	0.174
WC2				
R	11.2 (0.7)	13.6 (0.6)	6.350	0.018
E	10.5 (0.7)	12.1 (0.5)	3.894	0.060
T	10.8 (0.7)	13.0 (0.5)	6.436	0.018
CLS	96.2 (2.3)	114.8 (1.8)	40.83	<0.001

Numbers for TONI-4, CARS-2, and the CELF-4 subtests represent standard scores. *Numbers in parentheses* are standard errors of the mean. *P* and *F* values reflect a group comparison

*CFD* Concepts and Following Directions, *RS* Recalling Sentences, *FS* Formulated Sentences, *WS* Word Structure, *WC2* Word Classes, *R* receptive, *E* expressive, *T* total, *CLS* Core Language Score

**Table 3:** Group means for non-word repetition, auditory processing (TAPS-3) and ADHD symptoms (Conners’ Rating Scales)

	H-SLI	TD	*F*	*p*
Non-word repetition				
1 syllable	96.9 (1.3)	98.7 (0.7)	1.385	0.249
2 syllable	94.2 (1.0)	97.6 (0.8)	7.53	0.009
3 syllable	86.6 (2.7)	97.6 (0.7)	15.601	0.001
4 syllable	64.7 (3.3)	85.7 (2.1)	27.967	<0.001
TAPS-3 number memory				
Forward	7.3 (0.5)	10.9 (0.5)	23.069	<0.001
Reversed	8.9 (0.4)	11.9 (0.6)	16.04	<0.001
Conners’ ADHD Index	54.9 (2.4)	47.9 (1.3)	6.629	0.016

Numbers for TAPS-3 and Conners’ represent standard scores. Numbers for non-word repetition reflect percent correct of repeated phonemes. *Numbers in parentheses* are standard errors of the mean

In regard to language aptitude, while the H-SLI group’s CELF scores did not fall into the clinical range, they were nonetheless significantly lower than those of their TD peers for most of the administered subtests and for the cumulative CLS (see Table 2). Word Structure was the only CELF-4 subtest, on which the two groups did not differ. However, because the WS subtest is designed to be administered only to children younger than 9, this group comparison was based on a small number of participants (six H-SLI children and five TD children) and needs to be viewed with caution. At the individual level, nine H-SLI children scored 1 standard deviation or more below the mean on at least one subtest of CELF-4.

Lastly, the H-SLI children performed significantly worse on both the number memory forward and number memory reversed subtests of TAPS-3 and on the non-word repetition task (see Table 3). In the latter, the significant effect of group, *F*(1,36) = 38.089, *p* < 0.001, *η*_*p*_^*2*^ = 0.514, was further defined by a group by syllable interaction, *F*(3,108) = 12.662, *p* < 0.001, *η*_*p*_^*2*^ = 0.26, with H-SLI children being significantly less accurate at repeating 2-, 3-, and 4-syllable non-words. According to the study by Dollaghan and Campbell [23], scores of eight children on 4-syllable non-words were low enough to be three times more likely to come from children with language impairment than from children with typical language development.

### Experiment 1—audiovisual matching task

#### Behavioral results

Behavioral performance on the audiovisual matching task is summarized in Table 4. Overall, TD children were more accurate at matching heard words with silent articulations, *F*(1,36) = 10.708, *p* = 0.002. This effect was modified by a modest interaction with congruency, *F*(1,36) = 3.007, *p* = 0.091, *η*_*p*_^*2*^ = 0.077. Follow-up tests showed that TD children outperformed H-SLI children on both congruent, *F*(1,37) = 10.178, *p* = 0.003, and incongruent trials, *F*(1,37) = 7.995, *p* = 0.008. However, while H-SLI children were less accurate on incongruent than congruent trials, *F*(1,18) = 6.827, *p* = 0.018, *η*_*p*_^*2*^ = 0.275, their TD peers performed equally well on both, *F*(1,18) < 1. The two groups of children also had a small but significant difference in the number of misses (mean of 0.842 in TD vs. 2.342 in H-SLI; group, *F*(1,36) = 6.898, *p* = 0.013, *η*_*p*_^*2*^ = 0.161). Lastly, children’s RT was significantly shorter to congruent compared to incongruent trials—747 ms vs. 775 ms, respectively—showing the expected priming effect. This RT effect did not differ between groups: congruency by group, *F*(1,36) = 1.414, *p* = 0.242.

**Table 4:** Performance on the audiovisual matching task

	H-SLI	TD
Accuracy (% correct)		
Congruent	87.9 (1.7)	94.1 (0.8)
Incongruent	79.6 (4.2)	92.5 (1.7)
Reaction time		
Congruent	748.1 (37.0)	745.4 (33.4)
Incongruent	790.5 (41.2)	759.6 (33.2)
Misses		
Congruent	2.4 (0.6)	0.7 (0.2)
Incongruent	2.3 (0.6)	0.9 (0.3)

*Numbers in parentheses* are standard errors of the mean

While the lip-reading component of the audiovisual matching task was challenging for both H-SLI (mean 25.5 % correct, range 0–60 %, SD = 17.9) and TD children (mean 40.8 % correct, range 10–65 %, SD = 16.2), the TD children significantly outperformed their H-SLI peers, *F*(1,37) = 7.619, *p* = 0.009.

#### ERP results

##### Visual ERPs to the talker’s face and to pictures

**Fig. 3:**
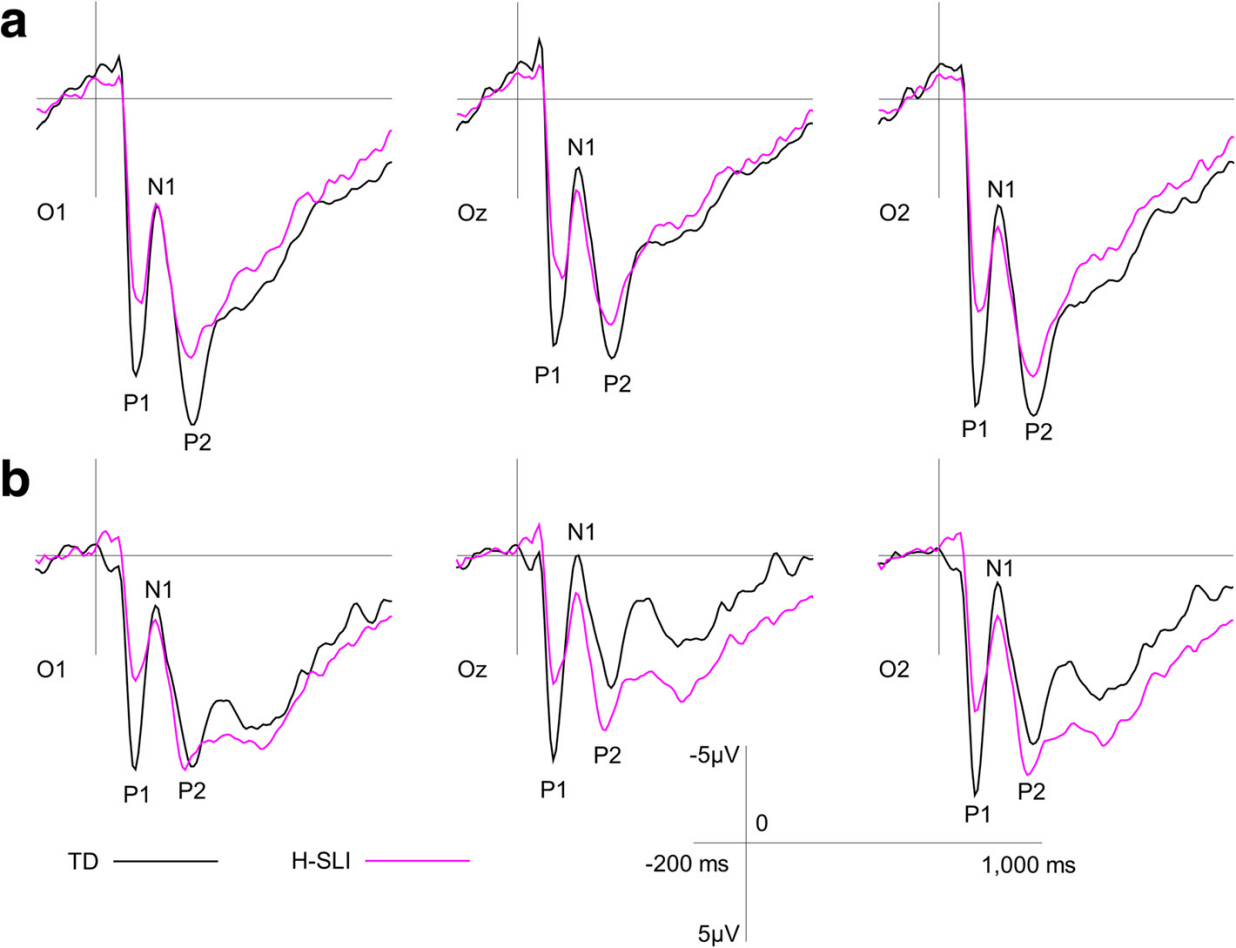
ERPs elicited by the appearance of the talker’s face (**a**) and by pictures accompanying auditory words (**b**). Grand average ERPs to the talkers face (**a**) and to pictures (**b**) are overlaid for the H-SLI and TD groups over occipital sites. Visual P1, N1, and P2 components are marked on each site. Negative is plotted up

ERPs elicited by both types of visual stimuli are presented in Fig. 3. The grand average waveforms show a clear sequence of the P1, N1, and P2 peaks. One TD child was excluded from the analysis of P1 to the talker's face because his peak amplitude measurement fell more than 2 standard deviations below the mean of either group. Our analyses of visual ERPs focused on the effect of group. The outcome of all comparisons is summarized in Table 5. There were two significant findings. First, the P1 peak amplitude to the talker’s face was significantly smaller in the H-SLI children compared to the TD group. Second, the P1 component elicited by pictures peaked significantly later in the H-SLI compared to the TD group.

**Table 5:** Group comparison of ERP components elicited by the talker’s face and by pictures

ERP component	H-SLI	TD	*F*	*p*	*η*_*p*_^*2*^
Talker’s face					
P1					
*Peak amplitude* (μV)	*15.61 (7.81)*	*22.52 (12.68)*	*4.489*	*0.041*	*0.114*
Peak latency (sec)	0.141 (0.017)	0.136 (0.016)	1.12	0.297	0.031
N1					
Peak amplitude (μV)	5.31 (8.91)	4.3 (13.84)	<1		
Peak latency (sec)	0.204 (0.018)	0.203 (0.017)	<1		
P2					
Peak amplitude (μV)	18.57 (10.1)	21.61 (11.17)	<1		
Peak latency (sec)	0.31 (0.025)	0.32 (0.026)	2.395	0.13	0.062
Pictures					
P1					
Peak amplitude (μV)	10.8 (7.77)	15.7 (10.11)	2.909	0.097	0.075
*Peak latency* (sec)	*0.14 (0.019)*	*0.128 (0.01)*	*7.763*	*0.008*	*0.177*
N1					
Peak amplitude (μV)	2.68 (7.43)	-0.318 (9.1)	1.377	0.248	0.037
Peak latency (sec)	0.203 (0.016)	0.202 (0.02)	<1		
P2					
Peak amplitude (μV)	14.49 (10.57)	13.39 (10.07)	<1		
Peak latency (sec)	0.313 (0.023)	0.322 (0.021)	2.105	0.155	0.055

Significant results are shown in italics. *Numbers in parentheses* are standard deviations

##### N400, LPC, and anterior negativity

**Fig. 4:**
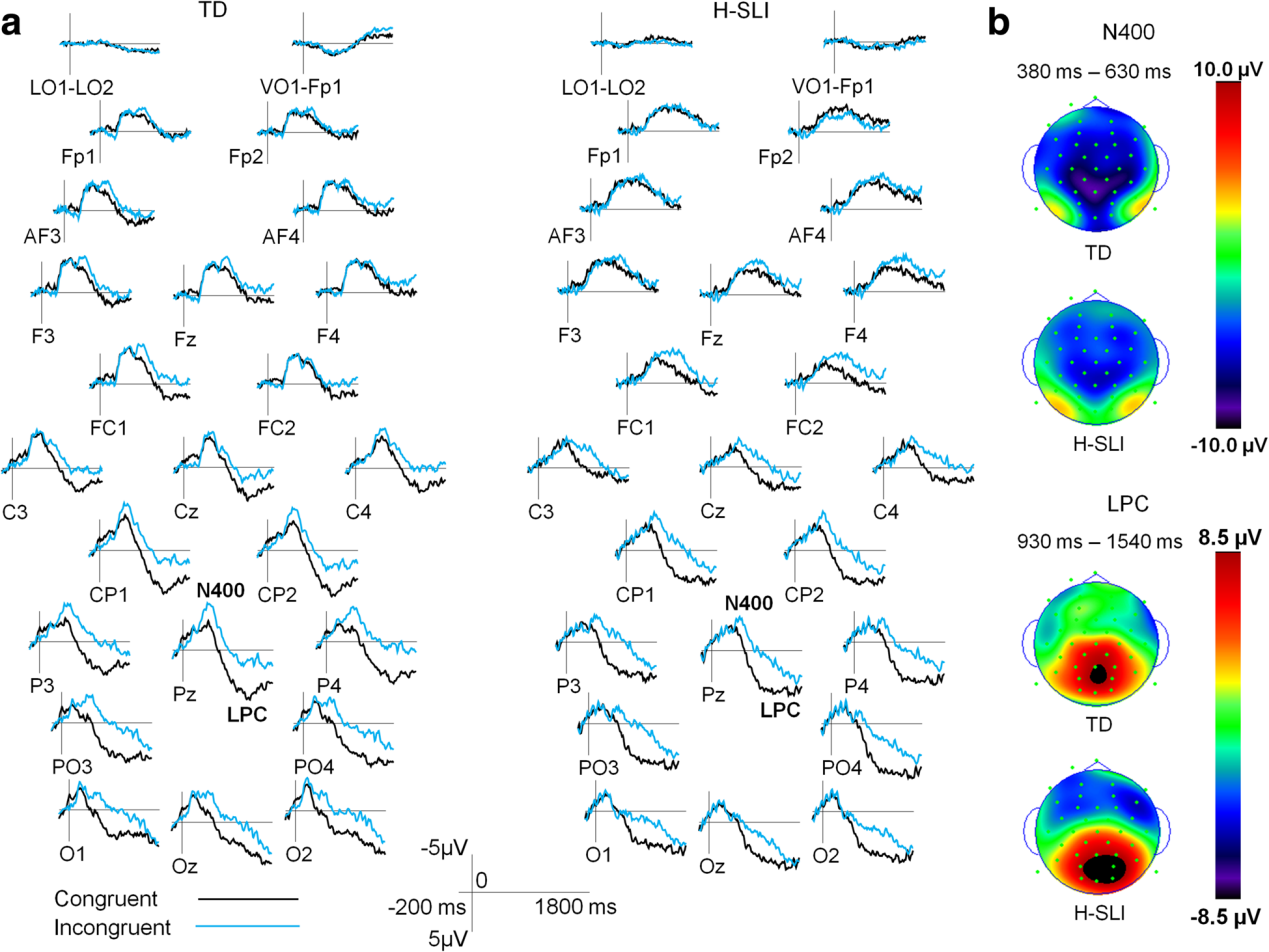
ERPs elicited by congruent and incongruent visual articulations—a within-group overlay. **a** Grand average ERPs elicited by congruent and incongruent articulations are overlaid separately for the TD and H-SLI children. The N400 and LPC components are marked on the Pz site. Negative is plotted up. **b** Voltage distribution measured as mean amplitude between 380 and 630-ms post-articulation onset is shown for the N400 component (*top*) and between 930- and 1540-ms post-articulation onset for the LPC component (*bottom*). Note the greater negativity in the TD group over the posterior scalp. While larger in absolute terms, the LPC in the H-SLI group was not significantly different from that in the TD group

**Fig. 5:**
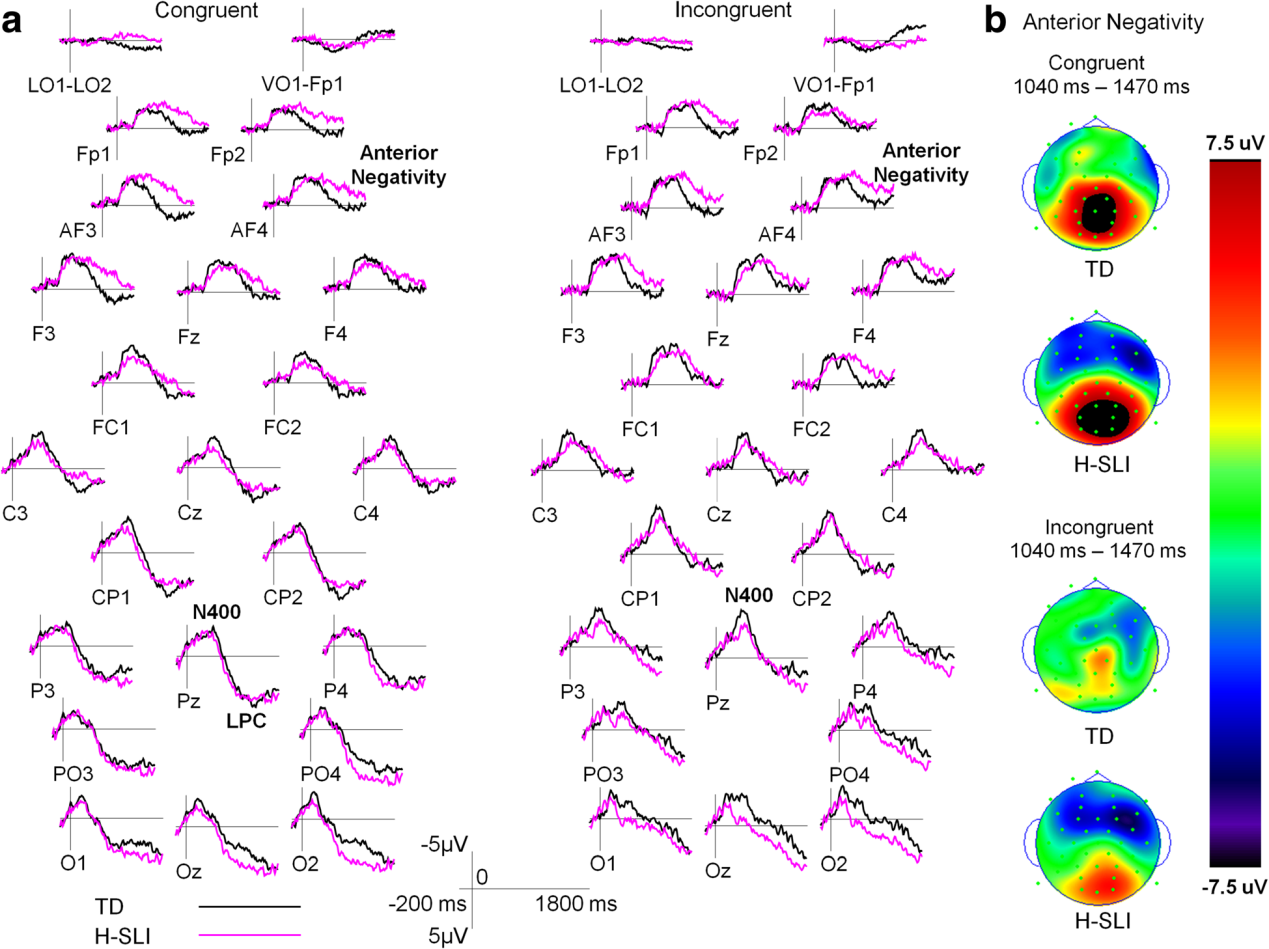
ERPs elicited by congruent and incongruent visual articulations—an across-group overlay. **a** Grand average ERPs elicited in the TD and H-SLI children are overlaid separately for congruent and incongruent articulations. The N400 and LPC components are marked on the Pz site. Anterior negativity is marked on the AF4 site. Negative is plotted up. **b** Voltage distribution measured as mean amplitude between 1040- and 1470-ms post-articulation onset is shown for the anterior negativity component on congruent (*top*) and incongruent (bottom) trials. Note significantly larger frontal negativity in the H-SLI group to both types of trials

Figure 4 overlays ERPs elicited by congruent and incongruent articulations in each group. Figure 5 contains the same data as Fig. 4 but allows for a more direct group comparison by overlaying ERPs of H-SLI and TD children to congruent (left side) and incongruent (right side) articulations. In conducting analyses of these components, we focused primarily on the effects of group, congruency, anterior to posterior distribution, and the interactions among these factors. The results are summarized in Table 6. Below, we provide a concise summary of main findings for each of the ERP components.

**Table 6:** Comparison of the N400, LPC, and anterior negativity mean amplitude in the H-SLI and TD groups

ERP component	Degrees of freedom	*F*	*p*	*η*_*p*_^*2*^	Direction
N400					
Group	1,36	1.863	0.181		
* Congruency*	*1,36*	*23.356*	*<0.001*	*0.393*	*incon > con*
* Group × congruency*	*1,36*	*4.635*	*0.038*	*0.114*	
Group in congruent	1,36	<1			
*Group in incongruent*	*1,36*	*4.517*	*0.04*	*0.111*	*TD > H-SLI*
*Anterior to posterior distribution*	*1,36*	*36.229*	*<0.001*	*0.502*	*CP,P > PO,O*
LPC					
Group	1,36	<1			
* Congruency*	*1,36*	*54.172*	*<0.001*	*0.601*	*con > incon*
Group × congruency	1,36	<1			
Anterior to posterior distribution	1,36	3.742	0.061	0.094	PO, O > CP, P
Anterior negativity					
* Group*	*1,36*	*4.43*	*0.042*	*0.11*	*H-SLI > TD*
Congruency	1,36	3.236	0.08	0.082	
Group × congruency	1,36	<1			
* Group × anterior to posterior distribution × site*	*1,36*	*3.837*	*0.058*	*0.096*	
*FP sites*	*1,36*	*5.913*	*0.02*	*0.141*	*H-SLI > TD*
*AF sites*	*1,36*	*6.803*	*0.013*	*0.159*	*H-SLI > TD*
F sites	1,36	3.358	0.075	0.085	H-SLI > TD
FC sites	1,36	1.417	0.242		
* Congruency × anterior to posterior distribution*	*1,36*	*16.167*	*<0.001*	*0.31*	
*F and FC sites*	*1,37*	*7.276*	*0.01*	*0.164*	*incon > con*
FP and AF sites	1,37	<1			

Significant results are shown in italics. *AF* anterior frontal, *CP* centro-parietal, *F* frontal, *FC* fronto-central, *FP* frontal polar, *P* parietal, *PO* parietal-occipital, *O* occipital; *con* congruent trials, *incon* incongruent trials

As expected, the N400 mean amplitude was significantly larger to incongruent compared to congruent articulations (see Fig. 4). The effect of congruency interacted with the group. Follow-up tests revealed that the N400 mean amplitude’s increase to incongruent articulations was smaller in the H-SLI compared to the TD children.^4^ The groups did not differ in the N400 amplitude to congruent articulations (see Fig. 5). Lastly, the N400 mean amplitude was overall larger in CP and P sites compared to PO and O sites.

The LPC mean amplitude was significantly larger to congruent compared to incongruent articulations. This effect did not interact with group. The LPC component was also marginally larger over the PO and O sites compared to the CP and P sites.

Incongruent articulations elicited greater anterior negativity than congruent ones over frontal and fronto-central sites. Additionally, there was a significant effect of group, with greater negativity in the H-SLI children. The effect of group was modified by a marginally significant interaction with anterior to posterior distribution and site. Follow-up tests confirmed that the H-SLI group had greater negativity compared to TD children over frontal polar and anterior frontal sites, with a similar trend over frontal sites. Groups did not differ over fronto-central sites. The group by congruency interaction was not significant.

### Experiment 2—speech-in-noise task

Both groups of children benefited significantly from seeing the talker’s face when listening to speech-in-noise (see Table 7): condition, *F*(1,34) = 544.233, *p* < 0.001, *η*_*p*_^*2*^ = 0.941. The effect of condition (A vs. AV) interacted with group, *F*(1,34) = 8.086, *p* = 0.007, *η*_*p*_^*2*^ = 0.192. Follow-up tests showed that while the two groups of children performed similarly in the A condition, TD children had significantly higher accuracy in the AV condition. Importantly, accuracy was not affected by the order of the A and AV sessions, *F*(1,34) < 1, and there was no group by condition by session order interaction, *F*(1,34) = 1.404, *p* = 0.244, suggesting that group differences in the AV condition were not due to differences in session order across the two groups.

**Table 8:** The pairing of auditory words and silent visual articulations. Note that articulations that are congruent (i.e., match the preceding auditory word) in list A are incongruent (i.e., do not match the preceding auditory word) in list B

	List A		List B	
	Auditory word	Silent visual articulation	Auditory word	Silent visual articulation
1	Shower	Shower	Candy	Donkey
2	Tree	Lamb	Cat	Cat
3	Jello	Jello	Jello	Monkey
4	Cat	Girl	Egg	Egg
5	Egg	Pool	Donut	Bottle
6	Donut	Donut	Zipper	Present
7	Zipper	Zipper	Donkey	Candy
8	Donkey	Donkey	Shirt	Shirt
9	Grapes	Grapes	Grapes	Farm
10	Police	Apple	Police	Police
11	Truck	Belt	Apple	Apple
12	Apple	Police	Truck	Truck
13	Monkey	Monkey	Monkey	Jello
14	Sandwich	Mailman	Sandwich	Sandwich
15	Car	Car	Car	Fish
16	Turtle	Turtle	Turtle	Popcorn
17	Squirrel	Squirrel	Squirrel	Pretzel
18	Window	Window	Window	Sandbox
19	Sled	Bird	Sled	Sled
20	Necklace	Necklace	Bread	Duck
21	Water	Water	Water	Carrot
22	Sink	Sink	Sink	Mop
23	Paint	Paint	Paint	Woods
24	Pretzel	Pretzel	Pretzel	Squirrel
25	Nail	Peas	Nail	Nail
26	Bird	Sled	Bird	Bird
27	Corn	Corn	Corn	Frog
28	Couch	Couch	Couch	Moose
29	Farm	Farm	Farm	Grapes
30	Airplane	Pumpkin	Airplane	Airplane
31	Popcorn	Popcorn	Popcorn	Turtle
32	Penguin	Doctor	Penguin	Penguin
33	Knife	Mouth	Mouth	Mouth
34	Arm	Horse	Arm	Arm
35	Bed	Ear	Bed	Bed
36	Present	Present	Present	Zipper
37	Sandbox	Sandbox	Sandbox	Window
38	Mop	Mop	Mop	Sink
39	Mailman	Sandwich	Mailman	Mailman
40	Lamb	Tree	Shower	Necklace
41	Candy	Candy	Duck	Bread
42	Scissors	Balloon	Scissors	Scissors
43	Pool	Egg	Pool	Pool
44	Bee	Bee	Bee	Eye
45	Chair	Boat	Chair	Chair
46	Cake	Ball	Cake	Cake
47	Boy	Boy	Boy	Dog
48	Sprinkler	Muffin	Sprinkler	Sprinkler
49	Elephant	Elephant	Elephant	Teddy bear
50	Comb	Beach	Comb	Comb
51	Jar	Purse	Jar	Jar
52	Horse	Arm	Horse	Horse
53	Sweater	Sweater	Sweater	Picture
54	Moose	Moose	Moose	Couch
55	Muffin	Sprinkler	Muffin	Muffin
56	Ear	Bed	Ear	Ear
57	Toys	Toys	Toys	Bus
58	Bus	Bus	Carrot	Water
59	Carrot	Carrot	Teacher	Buttons
60	Teacher	Teacher	Hammer	Hammer
61	Hammer	Pizza	Bus	Toys
62	Frog	Frog	Frog	Corn
63	Shirt	Foot	Necklace	Shower
64	Buttons	Buttons	Buttons	Teacher
65	Ball	Cake	Ball	Ball
66	Beach	Comb	Beach	Beach
67	Girl	Cat	Girl	Girl
68	Mouth	Knife	Knife	Knife
69	Peas	Nail	Peas	Peas
70	Woods	Woods	Woods	Paint
71	Picture	Picture	Picture	Sweater
72	Purse	Jar	Purse	Purse
73	Belt	Truck	Belt	Belt
74	Wolf	Wolf	Wolf	House
75	Scarf	Scarf	Scarf	Broom
76	Teddy bear	Teddy bear	Teddy bear	Elephant
77	House	House	House	Wolf
78	Eye	Eye	Eye	Bee
79	Dog	Dog	Dog	Boy
80	Flower	Orange	Flower	Flower
81	Doctor	Penguin	Doctor	Doctor
82	Foot	Shirt	Foot	Foot
83	Broom	Broom	Broom	Scarf
84	Tractor	Pencil	Tractor	Tractor
85	Circus	Money	Circus	Circus
86	Balloon	Scissors	Balloon	Balloon
87	Orange	Flower	Orange	Orange
88	Pencil	Tractor	Pencil	Pencil
89	Pumpkin	Airplane	Pumpkin	Pumpkin
90	Bread	Bread	Lamb	Lamb
91	Money	Circus	Money	Money
92	Bottle	Bottle	Bottle	Donut
93	Boat	Chair	Boat	Boat
94	Pizza	Hammer	Pizza	Pizza
95	Fish	Fish	Fish	Car
96	Duck	Duck	Tree	Tree

### Regressions

**Fig. 6:**
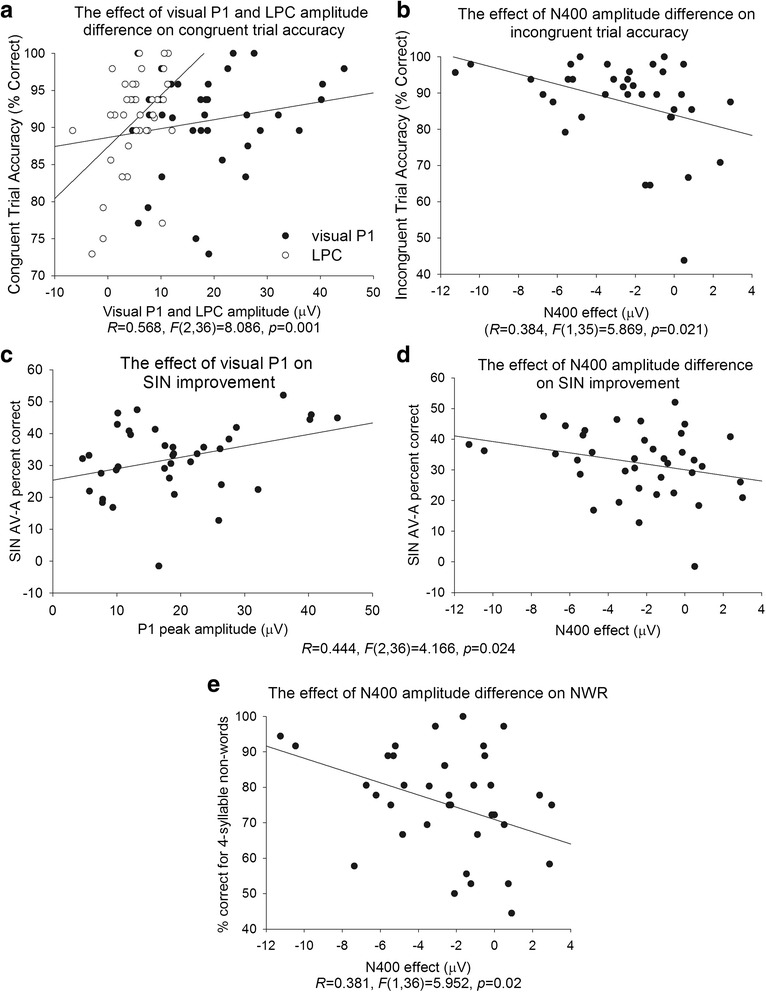
Linear regressions. In multiple regression analyses, ERP measures (the amplitude of visual P1 to the talker’s face, and the N400 and LPC differences between congruent and incongruent trials) were always entered as predictors, while behavioral measures were always entered as outcomes. Only significant results are shown. In cases where two predictors significantly determined behavioral outcomes, the provided statistic reflects the final model. The *top panel* shows relationships between ERP measures and performance on the audiovisual matching task. The *middle panel* shows relationships between ERP measures and SIN. Finally, the *bottom panel* reflects the relationship between ERP measures and performance on the non-word repetition task

Figure 6 visualizes significant regression results. Only one correlation between ERP measures and linguistic ability was significant—namely, larger N400 effect was associated with better accuracy when repeating 4-syllable non-words, *R* = 0.381, *B* = -1.785, *F*(1,36) = 5.952, *p* = 0.02. Two ERP measures were predictive of enhanced performance on the SIN task in the AV condition—namely, children with larger P1 and N400 showed the best improvement on the SIN when seeing the talker’s face, *R* = 0.444, *F*(2,36) = 4.166, *p* = 0.024. Finally, the peak amplitude of P1 and the LPC effect were both positively correlated with accuracy on *congruent* trials of the audiovisual matching task, with the final model accounting for approximately 32 % of variance, *R* = 0.568, *F*(2,36) = 8.086, *p* = 0.001. At the same time, the N400 effect was negatively correlated with accuracy on *incongruent* trials, with children who had larger N400s detecting the mismatch between the auditory word and the articulation more accurately, *R* = 0.384, *F*(1,35) = 5.869, *p* = 0.021.

We also conducted a linear regression between lip-reading skills and the SIN accuracy improvement in the AV condition. These two variables did not correlate, *F*(1,36) = 1.722, *p* = 0.198.

## Discussion

### Processing of visual articulatory movements in H-SLI children

The main task of the study probed how well children can associate a sequence of articulatory movements with a specific auditory word. Our results suggest that H-SLI children are less sensitive to auditory-articulatory correspondences. The significantly reduced P1 peak amplitude to the talker’s face and smaller N400 to incongruent articulations in the H-SLI group point to two possible causes of these children’s difficulty with the task.

First, the visual P1 component reflects exogenous influences on the visual system. It is sensitive to the sensory properties of visual objects, such as stimulus contrast. P1 reduction in the H-SLI group suggests that the early stage of visual processing may be less robust in these children. Visual processing in SLI has received significantly less attention than auditory processing. However, at least some previous studies did report a similar reduction of P1 to visual stimuli in SLI children (e.g., [68]). Although the two groups differed significantly in the P1 peak amplitude only to the talker's face, we do not believe that P1 attenuation was face-specific. It is worth noting that the grand average of P1 to pictures reflects sensory encoding of 48 different images while the grand average of P1 to faces reflects the encoding of just one image of the talker’s face. Therefore, the observed group differences may be driven by unique sensory properties of the talker’s face used in our study, rather than by faces as a category. Indeed, in electrophysiological studies of face processing, it is typically a later component—N170—that is sensitive to the presence of faces as compared to other visual objects (for a review, see [86]). Overall, the reduced amplitude of P1 to visual stimuli in children with a history of SLI suggests that more research focusing on the processing of complex visual information in SLI is needed.

However, an alternative (but related) interpretation of the P1 reduction in the H-SLI children is possible—namely, it may reflect poor attentional allocation to the visual stimuli. Although the role of attention in audiovisual processing is still a matter of debate, some studies suggest that when attention is diverted away from the visual stimulus or is taxed with an additional task, the influence of visual speech cues on auditory speech perception weakens [1, 103]. Importantly, we know that at least some aspects of attention are impaired in SLI. For example, selective [96] and sustained [29] attention to auditory stimuli has been shown to be atypical in SLI. More recent work in the visual domain shows that children with SLI have difficulty inhibiting visual distractors while attending to auditory words [109] and are slow to allocate attention to visual stimuli [22]. Numerous ERP studies of visual attention show that visual P1 is the earliest ERP component that can be modulated by attention (for reviews, see [41, 58]). Therefore, reduced attention to the talker’s face in the SLI group might have led to less robust sensory encoding of visual stimuli and, consequently, smaller P1.

Second, we interpret smaller N400 to incongruent articulations in the H-SLI group as a sign of imprecise auditory-articulatory correspondences in these children, at least at the level of individual phonemes and/or syllables. As we mentioned in the introduction, the study by Van Petten and colleagues [106] showed that N400 is elicited prior to the moment when a word can be reliably identified. This may be particularly true for visual articulatory presentations of words, which unfold over time, compared to printed words for example. Smaller >N400 to incongruent articulations therefore likely reflects difficulty with sub-lexical matching between auditory and articulatory information in children with a history of SLI. This finding is particularly striking because auditory word/articulation pairings on incongruent trials provided ample visual cues to the difference between the expected and the seen articulations (e.g., hear “sled,” see the articulation of “bird”). Although articulatory movements carry sufficient information about speech sounds to facilitate speech perception and even to differentiate different languages [83, 94, 111], this information is significantly less precise compared to the auditory signal in that multiple speech sounds will typically map onto the same observable articulatory gesture (e.g., sounds differing only in voicing ([b] vs. [p]) can be very difficult to differentiate based on observed articulation). The fact that the H-SLI children had difficulty even with extreme examples of auditory-articulatory mismatches suggests that they are likely even less sensitive to more subtle articulatory details that differentiate most speech sounds in English.

The N400 enhancement to incongruent articulations was significantly correlated with a number of behavioral measures. It strongly predicted performance on the audiovisual matching task itself. Additionally, larger N400 was associated with greater SIN accuracy improvement in the presence of the talker’s face. Because we used the same words as stimuli in the audiovisual matching and the SIN tasks, the same articulatory cues were available to children in both paradigms, and, as a result, a direct comparison between the two tasks is possible. This comparison suggests that those children who were less sensitive to auditory-articulatory mismatches in the audiovisual matching task were also less efficient at using visual articulatory cues when listening to speech-in-noise. This conclusion is supported by a significant positive correlation between accuracy on the audiovisual matching task and the degree of enhancement for SIN when seeing the talker’s face: incongruent trials vs. SIN, *r* = 0.286, *p*(one-tailed) = 0.045; congruent trials vs. SIN, *r* = 0.367, *p*(one-tailed) = 0.013. The relationship between the N400 amplitude elicited during audiovisual matching task and the SIN accuracy in children replicates an earlier finding from our laboratory, in which a similar correlation was found for adults [46].

Unlike the P1 and N400 components, the LPC elicited by congruent articulations was similar in TD and H-SLI children. A late latency of this component and its sensitivity to word repetition (e.g., [69, 74]) suggests that it reflects some aspect of word recognition. A significant correlation between the LPC effect and detection of congruent articulations in the audiovisual matching task supports this interpretation. The lack of a group difference in the LPC component is very informative. It suggests that H-SLI children’s deficit in audiovisual matching may be restricted to establishing auditory-articulatory correspondences at the sub-lexical (phonemic/syllabic) level. It also underlines the usefulness of online measures of visual processing in identifying the loci of audiovisual perception difficulties in this group.

Between approximately 1000 and 1500 post-stimulus onset, ERPs of the H-SLI group showed sustained negativity over the frontal scalp. This negativity was significantly smaller in the TD group, where it was present mostly in response to incongruent trials. The distribution of this component and its greater prominence to incongruent trials in the TD children suggests that it may be similar to the processing negativity described by Nӓӓtӓnen [66]. Typically, processing negativity is associated with selective attention paradigms, in which some stimuli are attended (and elicit more negative waveforms) while others are not. Within the context of our paradigm, greater sustained negativity in the H-SLI children may be a sign of greater effort required on their part to perform the task. This interpretation would agree with H-SLI children’s overall lower accuracy. Additionally, stronger anterior negativity may indicate that H-SLI children processed visual articulations for a longer period of time compared to their TD peers. According to this interpretation, larger anterior negativity to incongruent compared to congruent trials in the TD group might reflect a longer analysis of incongruent articulations, perhaps in an effort to understand the word being mouthed. The two interpretations are not mutually exclusive since any task that is more effortful may also require more time to complete.

### Study limitations

Our TD and H-SLI children were matched on age only. In the absence of a separate TD group matching H-SLI children on language skills, we cannot determine whether observed group differences reflect a true abnormality of audiovisual matching skills in H-SLI children or a maturational delay. Additionally, although the number of participants in each group of our study is typical for developmental electrophysiological studies and for studies of SLI in particular, power analyses suggested that our *n* was sufficient for detecting only large effects. Therefore, all reported negative outcomes should be interpreted with caution and require replication. Finally, differences in attention skills between TD and H-SLI children might have played a significant role in the outcome of the study. Although an adult assistant always stayed with children in the testing booth and redirected their attention to the task as needed, it is possible that the pattern of fixations on the talker’s face differed between the two groups. Indeed, recent work by D’Souza and colleagues shows that different developmental disorders may be associated with different patterns of face scanning [19]. Future studies that combine ERP recordings with eye-tracking may help determine whether H-SLI children differ from their TD peers in how they allocate attention to the talker’s face.

## Conclusions

The results of this study suggest that children with a history of SLI have poorly defined correspondences between speech sounds and observable articulatory movements that produce them. In broad terms, this finding shows that at least some of the processing and linguistic impairments characterizing SLI extend into the visual modality. Therefore, in order to have a more accurate picture of cognitive development in both typical and clinical populations, a better understanding of how different senses are combined in the developing brain is needed.

Our findings also have significance for a number of specific questions in SLI research and intervention. First, the mismatch between auditory and articulatory information in our stimuli was always present at the word onset. Word onsets are highly prominent parts of words [3, 33] and can be conceived as gateways to lexical access [34]. In most cases, articulatory movements precede the onset of sound in continuous speech ([17, 35, 61]; but see also [90, 107]). As a result, sensitivity to visual speech cues may facilitate lexical access by reducing the number of possible lexical items to be activated, particularly when listening conditions are poor. This facilitation may be reduced in H-SLI children.

Second, children with a history of SLI are at high risk for developing dyslexia [12]. Growing evidence suggests that this disorder is characterized by impairments in at least some aspects of audiovisual processing, such as audiovisual temporal function [38]. Hypothetically, the presence of audiovisual deficits in both disorders suggests that SLI children with greater audiovisual impairments might be at a higher risk for developing dyslexia. Because dyslexia is typically diagnosed later than SLI, the use of audiovisual screening measures with children with SLI might help identify individuals with higher risk for dyslexia before they start school. However, more work is needed to better understand audiovisual impairments characterizing both disorders.

Last but not least, our study shows that even when H-SLI children’s language scores on standardized tests do not fall below the clinical cut-off, their language and speech perception skills may still be remarkably different from those of their TD peers. Better understanding of the nature of language processing difficulties in this population, including audiovisual speech perception, may help provide these children and their families with better support.

## Appendix

**Table 7:** Group comparison on the SIN task

SIN condition	H-SLI	TD	*F*	*p*
A	41.4 (9.4)	39.2 (11.9)	<1	
*AV*	*69.3 (12.1)*	*76 (6.8)*	*5.02*	*0.032*

Numbers shown are percent of correctly identified words. *Parentheses* include standard deviations. Significant results are italicized

## Abbreviations

A: auditory
ADHD: attention deficit/hyperactivity disorder
AV: audiovisual
C&FD: Concepts and Following Directions
CARS-2: Childhood Autism Rating Scale (2nd edition)
CELF-4: Clinical Evaluation of Language Fundamentals (4th edition)
CELF-P2: Clinical Evaluation of Language Fundamentals Preschool (2nd edition)
dB: decibels
EEG: electroencephalographic
ERPs: event-related potentials
FS: Formulated Sentences
H-SLI: history of specific language impairment
LLI: language learning impairment
LPC: late positive complex
RS: Recalling Sentences
RT: reaction time
SIN: speech-in-noise
SLI: specific language impairment
SPELT: P2 should go with the abbreviation SPELT as follows: SPELT-P2, Structured Photographic Expressive Language Test—Preschool (2nd edition)
SPELT-II: Structured Photographic Expressive Language Test (2nd edition)
TAPS-3: Test of Auditory Processing Skills (3rd edition)
TD: typically developing
TONI-4: Test of Non-Verbal Intelligence (4th edition)
V: visual
WC-2 Total: Word Classes-2
WS: Word Structure

## Footnotes

1 There is significant variability in the terminology used to refer to children with SLI. Some researchers, like Knowland and colleagues, prefer the term “language learning impairment” in recognition of the fact that many of these children also have problems in other areas of cognitive development.

2 Because dyslexia is common among older children with a history of SLI, we did not exclude this child from our sample. However, as we describe in the “Discussion” section, even those children with dyslexia who never had language difficulties may still show some audiovisual deficits. To make sure that the child with dyslexia did not skew group comparisons, we repeated our analyses without this child’s data. Only one group comparison changed from significant to near significant (see the “Results” section).

3 All *t* tests that defined the boundaries of the anterior negativity window were significant. Five *t* tests that defined the boundaries of the LPC window were not. However, no more than 3 of non-significant *t* tests occurred in a row.

4 When the H-SLI child with dyslexia was excluded from the group analysis comparing the N400 to incongruent articulations, the effect of group fell just above the significance level at α = 0.05: *F*(1,35) = 3.928, *p* = 0.055, *η*_*p*_^*2*^ = 0.101. Although slightly reduced, the effect size measured by *η*_*p*_^*2*^ remained comparable (0.101 vs. 0.111). Based on DFBETA, the child with dyslexia was not an outlier in any of the regression analyses.
